# Trematodiasis occurrence in cattle along the Progo River, Yogyakarta, Indonesia

**DOI:** 10.14202/vetworld.2019.593-597

**Published:** 2019-04-22

**Authors:** Korbinianus Feribertus Rinca, Joko Prastowo, Dwi Priyo Widodo, Yudhi Ratna Nugraheni

**Affiliations:** Department of Parasitology, Faculty of Veterinary Medicine, Universitas Gadjah Mada, Yogyakarta, Indonesia

**Keywords:** cattle, *Fasciola* spp, identification, Indonesia, *Paramphistomum* spp, Yogyakarta

## Abstract

**Aim:**

This study aimed to measure the occurrence of trematodiasis in cattle along the Progo River, a district of Yogyakarta, Indonesia. The findings help to establish the magnitude of the disease and encourage prevention and treatment of this condition.

**Materials and Methods:**

Trematode eggs were extracted from 100 fecal samples collected from cattle. The eggs were examined using the sedimentation technique, and the method of Parfitt and Banks was used to differentiate *Paramphistomum* spp. eggs from *Fasciola* spp. eggs.

**Results:**

The infection rate of trematode parasites was 50%. Cattle experienced multiple infections of both *Paramphistomum* spp. and *Fasciola* spp., as well as single infections of one species or the other. All breeds were vulnerable to infections of both trematode species, although different cattle breeds, including Peranakan Ongole crossbreeds, Simmental crossbreeds, and Limousin crossbreeds, showed differences in infection rate. The highest rate of infection with *Paramphistomum* spp. (15.78%) occurred in the Simmental crossbreeds. The highest rate of infection (31.57%) with *Fasciola* spp. was in the Peranakan Ongole crossbreeds. Multiple infections of both *Paramphistomum* spp. and *Fasciola* spp. were highest in Simmental crossbreed cattle (28.97%).

**Conclusion:**

The high infection rates of trematode parasites found in fecal samples, particularly of *Fasciola* spp., indicate that the cattle along the Progo River in Indonesia experience a high rate of trematodiasis disease.

## Introduction

Commercial production of cattle in Indonesia faces various problems that require immediate attention. A major problem is disease, which directly affects animal health and reduces profits for cattle owners. Another increasing problem is economic losses at feedlots due to a reduction in the feed-conversion ratio and low distribution of body weight [1].

Of many factors that affect the economic value of cattle, an important one is infectious disease caused by parasites. Parasitic infections in animals can inhibit health, reduce growth, cause low birth weight, cause reproductive problems, and reduce the profitability of carcasses. Parasite disease is the main cause of economic losses in world livestock [2-4]. Economic losses due to parasites result from a reduction in the livestock population, reduced milk productivity, and reduced quantity of meat, as well as damage to some animal parts, such as the liver, that are infested with parasites and must be discarded [5]. *Paramphistomum* spp. and *Fasciola* spp. are the most common parasites that infect cattle in Indonesia, including in research locations, and no official measures are in place to eliminate trematodes from cattle in Indonesia. Trematodes and other parasites are responsible for heavy losses to livestock in Indonesia, hindering progress toward the government’s goal of self-sufficiency. It is necessary to research the trematodiasis incidence in Indonesia so that the data can form the basis for the local government to implement prevention and control policies regarding trematodiasis.

As noted, *Paramphistomum* spp. and *Fasciola* spp. are the main parasitic species causing disease in cattle in Indonesia [6,7]. Paramphistomiasis has appeared as a gastrointestinal disease in pets, causing high economic losses [8]. Fasciolosis in animals has a direct economic impact that is most generally experienced by breeders or owners in livestock sectors, due to the rejection of diseased animal parts such as livers, a reduction in animal productivity, a reduction in growth rate, and low calf birth weight for offspring of infected mothers [9]. The worldwide economic loss as a result of parasite infection, including fasciolosis in ruminants, is estimated to be US$3 billion/year [10]. No research has been conducted on trematodiasis in cattle reared along the Progo River, a special district of Yogyakarta, Indonesia, so this location was chosen for the present research.

This study aimed to measure trematodiasis in cattle along the Progo River. The data were intended to establish the magnitude of the disease and encourage preventive and treatment efforts.

## Materials and Methods

### Ethical approval

All procedures performed in this study were in accordance with the ethical standards of the Faculty of Veterinary Medicine, Universitas Gadjah Mada. No specific permissions were required. Samples were collected following standard procedures, without any harm to the animals.

### Sampling

A total of 100 fecal samples were collected from April 2018 to October 2018 from cattle reared along the Progo River. Feces were collected directly from cattle rectums. Samples were placed in plastic bags and brought to the Parasitological Laboratory at the Faculty of Veterinary Medicine, Gadjah Mada University, in a cooler. Fecal samples were then stored in a refrigerator at 4°C until further examination.

### Parfitt–Banks method

This method was used to determine the number of trematode eggs in cattle feces from 2-g fecal samples. Feces were placed in a mortar to which 20 mL of water was added. The mixture was filtered and included in the tube up to a height of 2 cm on the tube nozzle. The solution was allowed to stand for 15 min to form a precipitate. The clear liquid was removed and a precipitate 1 cm in height remained. Water was added to the remaining mixture in the tube up to a height of 2 cm on the tube nozzle, and then it was stirred and allowed to remain for at least 15 minutes, until a precipitate formed. The clear liquid was removed, up to three drops of 10% NaOH were added, and water was added to reach a height of 2 cm on the tube nozzle. The solution was made homogeneous by stirring and left for 15 min to form a precipitate. The clear liquid was removed; two drops of methylene blue were added, and the mixture was allowed to stand for some minutes. The solution of feces and methylene blue was poured into a petri dish for examination under the binocular microscope with a 4× magnification lens. If necessary, several milliliters of water were added to facilitate observation. Worm eggs were observed and documented. They were discriminated on the basis of color; *Paramphistomum* spp. appeared transparent and deep blue, while *Fasciola* spp. eggs were golden yellow in color [11]. Identification of trematode eggs was based on the literature [12].

## Results

The examination of feces following the method of Parfitt and Banks indicated a high rate of infection with trematode parasites among the 100 cattle sampled. The rate of infection of *Paramphistomum* spp. solely was 47% and that of *Fasciola* spp. solely was 48%, as shown in Table 1.

**Table-1:** Results of sedimentation test to determine the presence of *Paramphistomum* spp. and *Fasciola* spp. eggs in cattle feces.

Type of egg	Number of samples assessed	Number of samples that contained trematode eggs	Percentage of infection (%)
*Paramphistomum* spp.	100	47	47
*Fasciola* spp.	100	48	48

Cattle reared traditionally by the local people are the results of artificial insemination, which has produced some new breeds, such as crossbreeds between Ongole cattle and the local cattle, creating the Peranakan Ongole (PO) crossbreed; the Simmental crossbreed; and the Limousine crossbreed. Multiple infections of both *Paramphistomum* spp. and *Fasciola* spp. were found at a rate of 28.95% in Simmental crossbreeds, 23.25% in Limousine crossbreeds, and 21.05% in PO crossbreeds. Single infections of *Paramphistomum* spp. occurred at a rate of 15.79% in Simmental crossbreeds, 10.53% in PO crossbreeds, and 9.30% in Limousine crossbreeds. Single infections of *Fasciola* spp. occurred at a rate of 31.58% in PO crossbreeds, 7.89% in Simmental crossbreeds, and 4.65% in Limousine crossbreeds, as shown in Table 2.

**Table-2:** Results of Parfitt and Banks method to identify trematodes in cattle fecal samples.

Number of samples		Samples that contained trematode eggs				Percentage (%)			
		
Breed	n	*Paramphistomum* spp. + *Fasciola* spp.	*Paramphistomum* spp.	*Fasciola* spp.	Negative	*Paramphistomum* spp. + *Fasciola* spp.	*Paramphistomum* spp.	*Fasciola* spp.	Negative
PO crossbreed	19	4	2	6	7	21.05	10.53	31.58	36.84
Simmental crossbreed	38	11	6	3	18	28.95	15.79	7.89	47.37
Limousin crossbreed	43	10	4	2	27	23.25	9.30	4.65	62.8

PO: Peranakan ongole

## Discussion

The rate of trematodiasis in cattle in the Progo river flow zone reaches 50%. This rate is higher than the rate of trematodiasis reported in Central Sulawesi, Indonesia (38.31%) [13], and the rate of infection with multiple trematode species is higher than in Malaysia (10%) [14]. These high rates of infection with both species might be caused by ineffective treatment, the immune response of different host species, cattle rearing management, differences in animal species and breeds, differences in the parasite measurement technique, differences in geography or climate, variations in ecological conditions, and the presence of intermediary hosts. At the study site, *Paramphistomum* spp. and *Fasciola* spp. were found living in watery, swampy areas that are suitable for the development and reproduction of the intermediary host, resulting in high prevalence [15].

The rate of *Paramphistomum* spp. infection in cattle in the Progo river flow zone was 47%, higher than in a previous study [16]. The rate of trematodiasis was 4% in Central Java, Indonesia; 12.1% in Aljazair [17]; 12% in northern Portugal and northwest Spain [18]; 18.8% in Galicia (Spain) [19]; 7% in northwest Uruguay and northwest Spain [20]; and 18.8% in Ethiopia [21]. The infection rate in the present study was higher than in a study conducted in northwest Ethiopia, where it was 45.85% [22]. These differences may be affected by factors such as differences in sample size, diagnostic technique, climate, ecology, and livestock management system [23].

The rate of *Fasciola* spp. infection reached 48%, higher than in previous studies that found rates of 3.28% in Iran [24] and 22.17% in Bangladesh [25]. However, it was lower than reported by Ashraf *et al.*, indicating that *Fasciola* spp. infection in cattle in tropical areas ranged from 30% to 90% [26]. Other studies reported *Fasciola* spp. infection rates of 36% in Sobangan Bali [27], 33.33% in Egypt [28], and 18% in Australia [29]. The difference in incidence between areas might be caused by differences in livestock rearing method, number of total samples, cattle composition, biological potentials of intermediary host and host, climate and topography of sampling location, metacercariae resistance in the environment, and diagnostic technique [30,31].

Infection rates of *Paramphistomum* spp. and *Fasciola* spp. of various cattle breeds in the present study were highly affected by rearing management. A previous report from Japan [32] showed that the rate of fasciolosis was higher in cattle breeds originating from Japan than in Friesian and Jersey breeds, due to management factors. Friesian breeds were not pastured on grassland, whereas Japanese breeds were pastured on natural grassland wet fields and were able to feed on rice straw, resulting in a higher rate of fasciolosis. Feeding rice straw also affected the rate of infection of *Paramphistomum* spp. The metacercaria on rice stalks are generally distributed along the bottom third of the rice stalk, so it is recommended to provide only the top half of the rice straw to cattle. In addition, using a grass drying method helped to prevent parasitic infection because exposure to direct sunlight for 2–3 days killed the metacercaria [33].

Malnutrition in animals also increases their vulnerability to parasitic infections. Poor animal health also reduced cattle’s resistance to infection challenges. Infection with *Fasciola* spp. was higher in cattle that had poor body condition scores (BCS). Many studies have indicated that there is a positive correlation between a reduction in cattle body weight and fasciolosis. The prevalence of fasciolosis was 85.9% for those in poor condition, 55.1% for those in medium weight, and 34.5% in those with good body weight [34]. A previous study by Jaja *et al*. [35] reported a significant correlation between BCS and infection intensity, suggesting that poor body condition might be a direct result of fasciolosis pathogenesis. Considering the important role of homeostasis in the liver and all metabolisms of animals, loss of BCS in infected cattle could be a result of *Fasciola* infection and might be correlated with metabolic disorders [36].

## Conclusion

The data from the present study indicated that the infection rate with multiple trematode parasites was highest in PO crossbreed cattle, whereas the percentage of *Paramphistomum* spp. infection alone was highest in the Simmental crossbreeds. These results should be applied to the development of a management plan to reduce the incidence of trematodiasis in this region.

## Authors’ Contributions

DPW, YRN, DPW, and JP designed the study. KFR and YRN conducted the field survey, collected samples, and examined them in the laboratory. All authors drafted and revised the manuscript. All authors read and approved the final manuscript.
